# Metacommunity analysis of amoeboid protists in grassland soils

**DOI:** 10.1038/srep19068

**Published:** 2016-01-11

**Authors:** Anna Maria Fiore-Donno, Jan Weinert, Tesfaye Wubet, Michael Bonkowski

**Affiliations:** 1 Institute of Zoology, Department of Terrestrial Ecology, University of Cologne, Cologne, Germany; 2 UFZ - Helmholtz Centre for Environmental Research, Department of Soil Ecology, Halle (Saale), Germany; 3 German Centre for Integrative Biodiversity Research (iDiv) Halle-Jena-Leipzig, Leipzig, Germany

## Abstract

This study reveals the diversity and distribution of two major ubiquitous groups of soil amoebae, the genus *Acanthamoeba* and the Myxomycetes (plasmodial slime-moulds) that are rarely, if ever, recovered in environmental sampling studies. We analyzed 150 grassland soil samples from three Biodiversity Exploratories study regions in Germany. We developed specific primers targeting the V2 variable region in the first part of the small subunit of the ribosomal RNA gene for high-throughput pyrotag sequencing. From ca. 1 million reads, applying very stringent filtering and clustering parameters to avoid overestimation of the diversity, we obtained 273 acanthamoebal and 338 myxomycete operational taxonomic units (OTUs, 96% similarity threshold). This number is consistent with the genetic diversity known in the two investigated lineages, but unequalled to date by any environmental sampling study. Only very few OTUs were identical to already known sequences. Strikingly different OTUs assemblages were found between the three German regions (PerMANOVA p.value = 0.001) and even between sites of the same region (multiple-site Simpson-based similarity indices <0.4), showing steep biogeographical gradients.

Protists play critical roles in soils, mainly as bacterial grazers, stimulating the rates of organic matter decomposition^1^ and shaping bacterial communities^2^,^3^,^4^. Our current understanding of soil protistan diversity and function is limited by our ability to precisely identify and quantify free-living species. Inventories of protists in soils mostly rely on the Most Probable Number method (see^5^ and citations therein). This approach has a strong bias towards cultivable organisms and underestimates species richness. Generally only broadly defined morphological groups are identified, i.e. flagellates, ciliates, naked or testate amoebae^6^,^7^ that are polyphyletic assemblages, interspersed throughout various eukaryotic supergroups^8^. The relative proportions of these assemblages vary greatly between studies, though naked amoebae are found to be one of the major morphotypes in soil^5^, and perhaps even the most abundant^9^,^10^.

Environmental sequencing offered potential new insights into biodiversity, especially in aquatic environments, with less attention given to terrestrial ecosystems. In the latter, most studies have focussed on fungi, microfauna and prokaryotes^11^,^12^,^13^,^14^, leaving the protists in limbo. Molecular biology of protists is challenging on its own - the genetic divergence observed between and within major protistan groups greatly exceeds that between animals, plants and fungi^15^. This has the direct and unfortunate consequence that molecular markers are strongly biased towards only a few lineages^16^,^17^. Most striking is the underrepresentation of the phylum Amoebozoa (including a substantial portion of the “naked” and “testate” amoebae) in all studies conducted using various “universal eukaryotic primers”^17^,^18^,^19^,^20^,^21^,^22^. In contrast, RNA-centred transcriptomic approaches (without a primer-based amplification step) identified Amoebozoa as one of the major terrestrial protist groups^23^,^24^.

The class Myxomycetes includes ca. 950 described species, but is poorly represented in existing databases, with only 101 SSU sequences currently available^15^. In contrast, *Acanthamoeba is* a genus of ca. 20 described species that is well represented in SSU databases by 1240 sequences^15^. Myxomycetes (also called Myxogastria) have a complex life-cycle, including amoebae and flagellate cells, a multinucleate plasmodium that can reach conspicuous dimensions and often macroscopic fruiting bodies bearing spores.

World wide inventories have been established based on the systematic collection of such fruiting bodies (listed in http://eumycetozoa.com/data/genera.php, updated June 11, 2014)^25^. Because they have been classified as fungi for a long time, Myxomycetes are excluded from amoebozoan treatises^26^ and consequently from most soil inventories^5^. Providing that specific molecular probes are used, dark-spore Myxomycetes can be recovered from soils^27^,^28^,^29^, litter^30^ and air^31^. This class benefits from a well-studied phylogeny^32^,^33^,^34^,^35^, with a major division between the dark spore and bright spore clades, the former being the better studied to date and the group targeted here.

*Acanthamoeba* is an abundant and ubiquitous genus in soil^9^,^36^, and some species are opportunistic human pathogens. Its classification is mainly based on cyst morphology^37^, defining three major groups. Molecular phylogenies confirmed the monophyly of a basal group I, highly divergent from the remaining 18 SSU rDNA genotypes of *Acanthamoeba*^38^,^39^,^40^,^41^,^42^. In contrast, the remaining two groups are phylogenetically related and even intermingled. The genotypes sometimes contain several different morphotypes and species, in disagreement with current classification^43^; on the other hand, some morphologically identical strains belong to different genotypes^36^, showing a need for systematic revision.

We investigated these two groups of amoebae in the Biodiversity Exploratories located in three regions in north-eastern, central and south-western Germany^44^. The Exploratories include a standardized network of study plots along a gradient spanning from semi-natural to intensively managed grasslands, where soil samples have been jointly collected and shared between research groups to explore multiple domains of biodiversity (www.biodiversity-exploratories.de, last accessed 30 June 2014).

Our aim was to characterize the biodiversity and the distribution of two amoebozoan taxa, the dark spored Myxomycetes and the genus *Acanthamoeba*, because of the growing evidence of their prevalence in soils and because they are consistently overlooked by both traditional and molecular sampling methodologies.

## Results

We obtained 177,282 sequences (Table 1) by filtering out 81% of the nearly one million reads by applying very stringent filtering parameters to avoid overestimation of protist diversity due to sequencing errors^45^. By de-replication, we obtained 5,722 clusters. Using BLAST, 87% of these could be assigned to *Acanthamoeba* or Myxomycetes reference sequences. The Silva database was not appropriate to identify Myxomycetes, since it missed 64% of the Myxomycetes OTUs. We found almost 7-fold more chimeras in the genetically more diverse Myxomycetes (47%) than in *Acanthamoeba* (7%). In the end 611 OTUs (representing 154,645 sequences) were obtained for the three German Biodiversity Exploratories, ie Schorfheide-Chorin (SEG), the National Park Hainich (HEG) and the Biosphere Reserve Schwäbische Alb (AEG) taken separately (Table 1). With the three sites analyzed together, 415 OTUs were obtained (192 *Acanthamoeba* and 223 Myxomycetes). Our primers targeting the V2 variable region in the first part of the small subunit of the ribosomal RNA gene were highly specific, resulting in only 9% of non-Myxomycetes or non-*Acanthamoeba* sequences (Table 2) while 4% of sequences did not match any known sequence.

### Taxonomic assignment by BLAST and phylogeny

**Figure 1:**
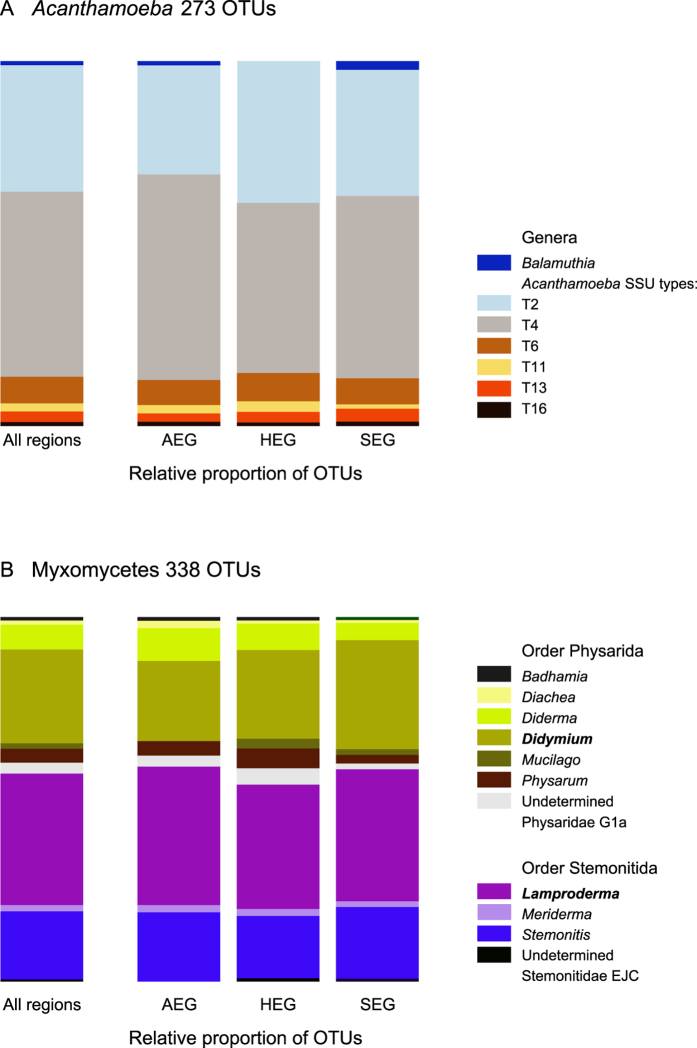
Relative contribution of the OTUs (96% similarity cut-off) to the taxonomic diversity. Taxonomical assignment is based on the best hit by BLAST. (**A**) *Acanthamoeba*, one related genus, *Balamuthia* has been found in addition to the genotypes T1-T16. (**B**) Myxomycetes, ten genera and two undetermined taxa, in two orders. The two dominant genera are in bold.

The 273 obtained OTUs for *Acanthamoeba* represented 36 unique BLAST hits (Table 1, Fig. 1A). Six of the 18 described acanthamoebal genotypes were recovered, plus a few OTUs from *Balamuthia mandrillaris,* a related genus used as an outgroup. The majority of reads were assigned to *Acanthamoeba* genotypes T4 and T2, but T6, T11, T13 and T16 were represented as well. We did not recover representatives from the Group I and from *Acanthamoeba lenticulata* and related genotypes.

Phylogenetic analyses, based on a short fragment, led to a mostly unresolved tree in its basal branching, supporting only few monophyletic groups with confidence (Fig. S1). It mostly confirmed the taxonomic assignment by BLAST, but also revealed the existence of two new clades of *Acanthamoeba*. These were composed of two and four OTUs and characterized by two strikingly different structures of the helix 10 that did not match any known sequence. Three of these sequences were assigned by BLAST to T4, one to T2 and one to T6, on the basis of the sequences of the helix 10e1 that differed between them. This suggests that these sequences might be chimeric. We decided to keep them in our analyses because these two new structures of the helix 10 likely represent two novel clades of *Acanthamoeba* (Fig. S1).

The 338 OTUs of Myxomycetes represented 35 unique BLAST hits (Table 1, Fig. 1B). Ten myxomycete genera and two undetermined taxa were retrieved, representing both dark-spored orders, Physarida and Stemonitida. The most abundant OTUs belonged to the genera *Lamproderma* (Stemonitida) and *Didymium* (Physarida) (Fig. 1B). Phylogenetic analysis lead to a tree showing Stemonitida paraphyletic to the monophyletic Physarida (66 to 85% support). The majority of the OTUs assigned to *Lamproderma* belonged in a clade with two reference sequences of *L. scintillans*, suggesting that *L. scintillans* is probably a species complex (Fig. S2). *Protophysarum phloiogenum*, a Physarida, was mis-assigned to Stemonitida, probably due to a long-branch attraction artefact. For both *Acanthamoeba* and Myxomycetes, we provide a complete list of the OTUs, their taxonomic affiliation according to BLAST and their occurrences in each soil sample (Table S4).

### Hidden diversity and rarefaction curves

**Figure 2:**
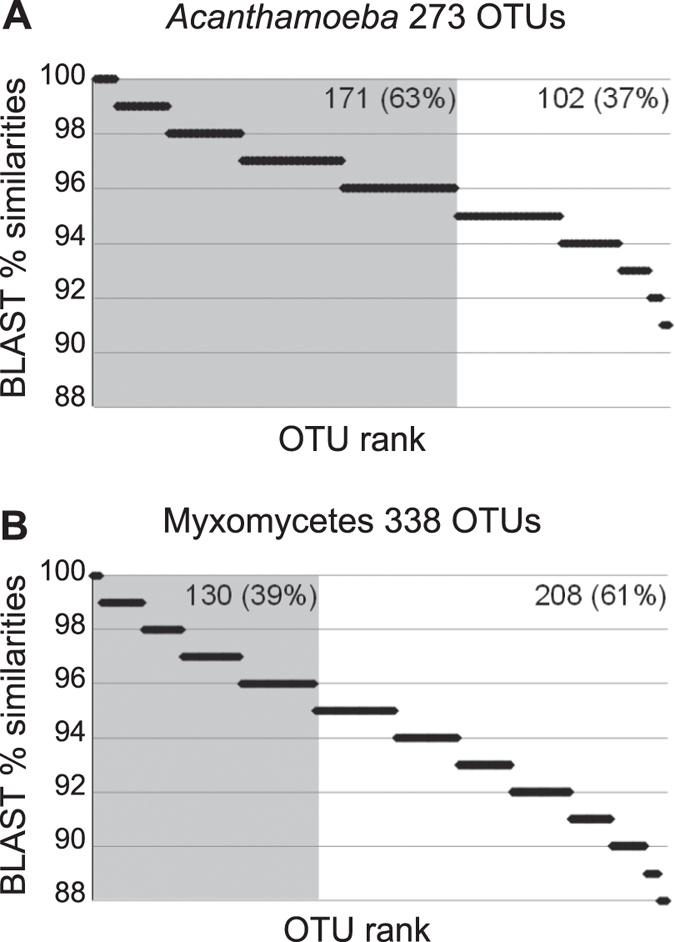
Similarities of the OTUs with known sequences. OTUs are classified according to their percentage of similarity to the next kin by BLAST. Horizontal bar size is proportional to the number of OTUs in each rank. Grey shadow highlights OTUs with ≥96% similarity: (**A**) *Acanthamoeba*; (**B**) Myxomycetes.

**Figure 3:**
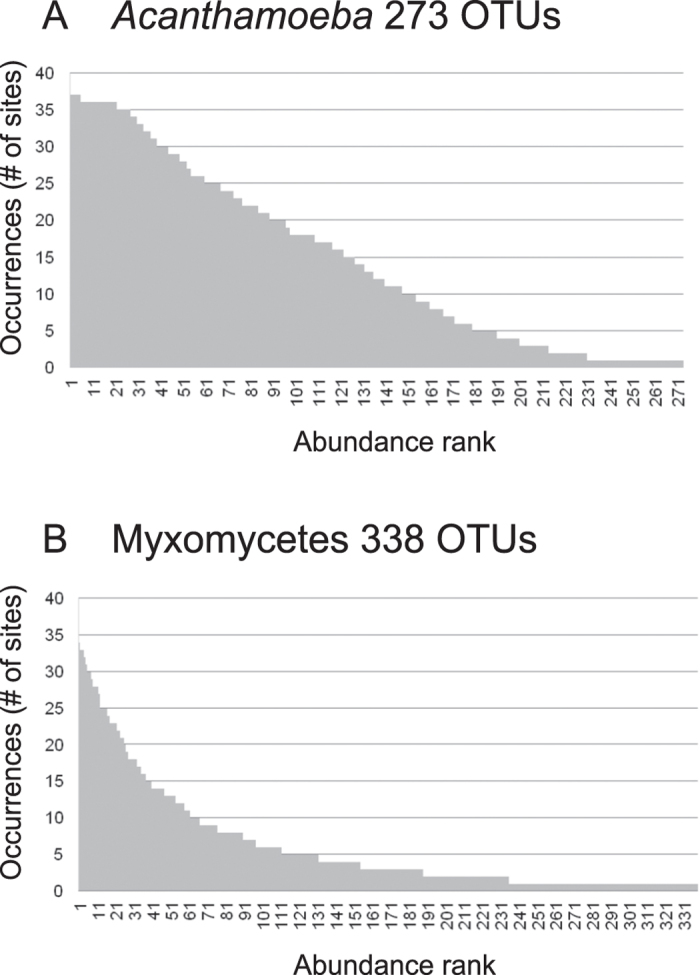
Rank abundance histogram of the OTUs. (**A**) In *Acanthamoeba*, OTUs abundance decreases regularly, showing few rare OTUs. (**B**) In Myxomycetes, few OTUs are abundant and many are rare.

**Figure 4:**
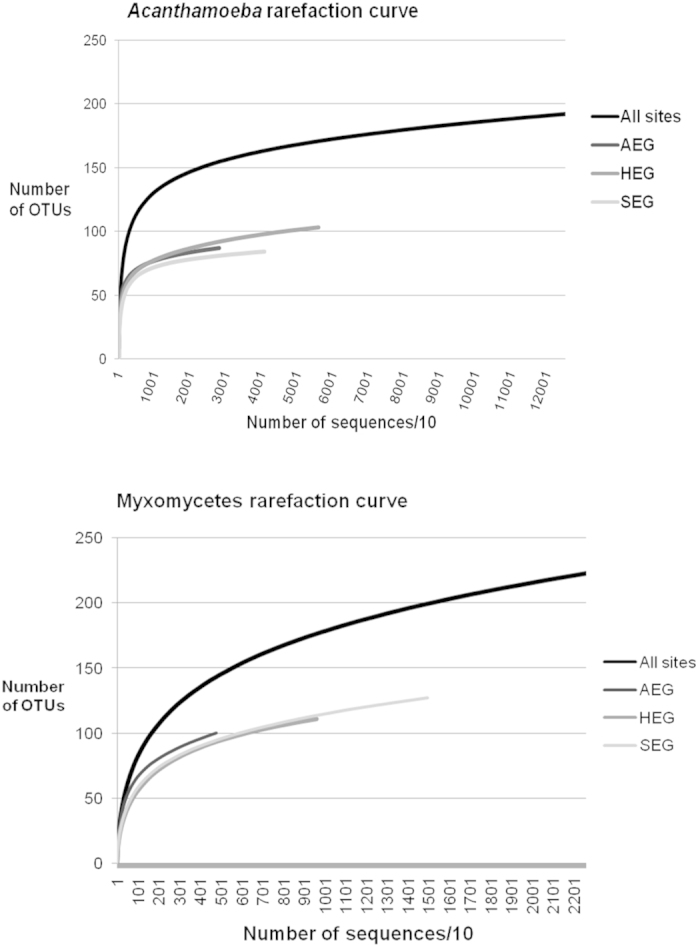
Rarefaction curves calculated for the entire dataset (All sites) and for each region (AEG, HEG and SEG). The number of OTUs in all regions together is smaller than the total number of OTUs, because some are shared between regions.

Only 39% of the myxomycete OTUs were 96–100% similar to any known sequence. For the genus *Acanthamoeba,* with a twelve fold larger reference database, 63% of the sequences spanned the same interval (Fig. 2). There were however no *Acanthamoeba* OTUs below 91% similarity (Fig. 2A), while 11% of the myxomycete OTUs were in the interval of 88 to 91% similarity to any known sequence (Fig. 2B). Rank-abundance curves of the OTUs showed very different patterns for *Acanthamoeba* and Myxomycetes, with less rare species in the former than in the latter (Fig. 3). Rarefaction curves, based on the total number of OTUs and sequences, suggested that sequencing depth was not sufficient for both groups, although in a lesser extent for *Acanthamoeba* (192 OTUs resampled from 125,316 sequences) (Fig. 4A), than for Myxomycetes, where more sequencing would have revealed more diversity (223 OTUs resampled from 29,329 sequences) (Fig. 4B). The Chao1 richness estimator predicted 226 OTUs for *Acanthamoeba* (95% upper bound: 277, lower bound: 205) and 287 OTUs for Myxomycetes (95% upper bound: 347, lower bound: 256), accordingly 69–94% of the acanthamoebal and 64–87% of the myxomycete extant diversity was recovered.

### Beta diversity

**Figure 5:**
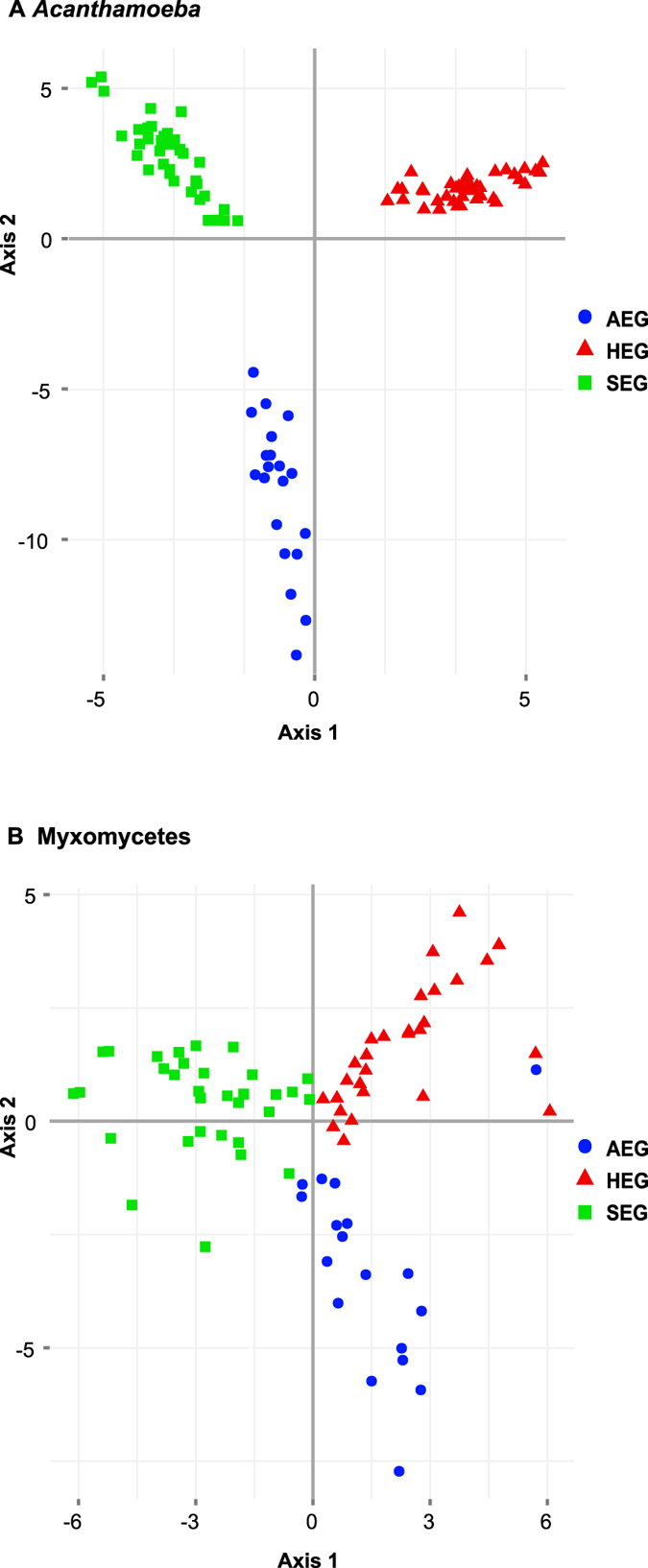
Inter-region similarity: Principal coordinates analysis (PCoA) ordination based on Hellinger-transformed OTUs abundances of the three exploratories showing a clear biogeographic distribution. Rare OTUS (<10 sequences) were removed, leaving 208 OTUs (124 *Acanthamoeba* and 84 Myxomycetes) (**A**) *Acanthamoeba*. (**B**) Myxomycetes.

**Figure 6:**
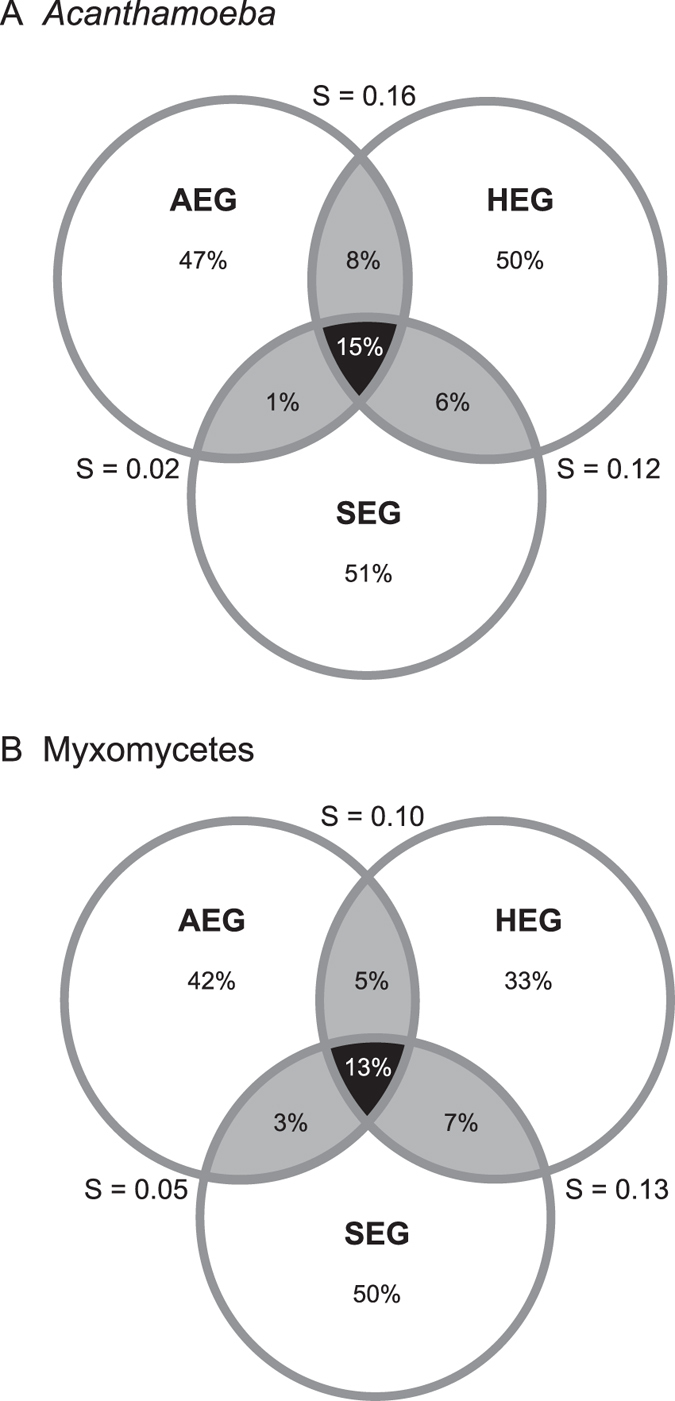
Inter-region similarity: Percentages of unique OTUs per region (in the circles) and shared between regions (in the intersections). Shaded in black, OTUs shared by the three regions (as percentage of the sum of the three regions); shaded in grey, OTUs shared between two regions (percentage of the sum of the two regions). S, Sørensen similarity index between two regions. (**A**) *Acanthamoeba*, total richness = 192. (**B**) Myxomycetes, total richness = 223.

OTUs were recovered from nearly all 146 sites that yielded amplicons: *Acanthamoeba* OTUs were found in 136 sites (93%) and Myxomycetes in 134 (92%) (*Acanthamoeba* AEG: 39, HEG: 48, SEG: 49; Myxomycetes AEG: 45, HEG: 45, SEG: 44) (Fig. S3 and Table S4). The most striking finding was that the three exploratories harboured surprisingly distinct OTUs assemblages (PerMANOVA, R^2^ = 0.18 for *Acanthamoeba*, R^2^ = 0.06 for Myxomycetes, p = 0.001 for both), although the few shared OTUs represented a high percentage of the total sequences (*Acanthamoeba*: 87%, Myxomycetes: 91%). Both communities, *Acanthamoeba* (r^2^ = 0.925, P < 0.001) and Myxomycetes (r^2^ = 0.669, P < 0.001) were well separated by PCoA between the three regions (Fig. 5), even when we removed the OTUs with less than 10 sequences, that is, with only 208 OTUs (124 *Acanthamoeba* and 84 Myxomycetes) (Table S4). The analysis of similarities suggested that the variation was evenly distributed between regions and within sites, the anosim R values being close to zero (>0.05 < 0.45 p.values < 0.005). Only 15 and 13% of OTUs in *Acanthamoeba* and Myxomycetes, respectively were shared between the three regions (Fig. 6), AEG and SEG having less OTUs in common (16% for both taxa) (Fig. 6). Inside each exploratory, the infra-site diversity was low (Table 3), but similarity was higher for the less-diverse *Acanthamoeba* than for Myxomycetes.

Nestedness was found for both taxa, *Acanthamoeba* and Myxomycetes, between and within regions (Table 4). The NODF metric (decreasing fill and paired overlap) allows unveiling if the nestedness results from differences between sites or among species^46^. *Acanthamoeba* and Myxomycetes tended to display nestedness in both species composition and site occupancy (Table 4).

## Discussion

Our study, the first to target amoebozoans in soils at such a scale, revealed that *Acanthamoeba* and dark-spore Myxomycetes communities are widely distributed, highly diverse and non-randomly structured in grassland soils. Most importantly, we found strikingly distinct communities between the three studied regions, the two investigated taxa showing clear biogeographic patterns of distribution, in particular *Acanthamoeba* (Fig. 5). We found a diversity level unequalled to date, with *Acanthamoeba* dominated by genotypes T4 and T2 and Myxomycetes by the genera *Lamproderma* and *Didymium* (Fig. 1), but also unlisted OTUs and possibly two new clades of *Acanthamoeba*.

*Acanthamoeba* has been used as a model organism for investigating biotic interactions in the rhizosphere. Indirect or direct effects, as observed in microcosm experiments, include enhanced plant growth^47^, changes in the root architecture^48^, shifts in the bacterial community structure by selective grazing^49^. Our study indicates that *Acanthamoeba* is present in such abundance and diversity that it may play a major ecological role in grassland ecosystems by the above cited interactions. For Myxomycetes, grassland is not considered as an obvious habitat^50^, despite evidence from MPN studies showing that amoeboflagellates were more common there than in forest soils^51^,^52^. Because some species are also fungivores^53^ Myxomycetes may play an unsuspected role in the bacterial-fungal dynamics in grassland ecosystems, and *Didymium*, because it is easily cultivable and common in soil, should be considered as model organism.

Our study upholds previous evidence that Amoebozoa is a major group of soil protists, as indicated by direct observations^36^,^54^ and from metagenomic approaches^23^,^24^. This provides strong evidence that the absence of Amoebozoa in general and Myxomycetes in particular from environmental sequencing studies may be only artefactual. The most likely explanation for the failure to find Myxomycetes in molecular environmental DNA sampling is their unusually long SSU rDNA gene and extremely divergent sequences^32^,^55^. The highest number of protistan soil environmental OTUs to date (1,014, 97% similarity threshold) was obtained by Bates *et al.* (2013) from 40 soil samples. Thirteen protistan phyla were recovered, but amoebozoans were only marginally represented, probably because the primers F515 and R1119 miss 67% of the amoebozoan and 100% of the myxomycete sequences of the Silva non-redundant SSU rDNA reference dataset. In addition, we have shown here that myxomycete environmental sequences may be misidentified if compared with the Silva dataset or may be discarded as not alignable. To compensate for that, we have provided a dark spore myxomycete reference dataset for BLAST purposes (Supplementary information 5) and SSU rDNA gene V2 template alignments for *Acanthamoeba* and Myxomycetes (Supplementary information 6 and 7).

Our results confirm that Myxomycetes are much more widespread than the occurrence of the ephemeral fruiting bodies suggests. We could probably only recover a small subset of a much larger assemblage, as shown by the proportion of sequences with low similarity to known sequences (Fig. 2B, Fig. S2), by the prevalence of rare OTUs (Fig. 3B), and by the slope of the saturation curve (Fig. 4B). Little is known about the abundance of Myxomycetes in soils. Inventories based on MPN methods indicated *Didymium* as the most common genus^56^,^57^, but we observed a predominance of *Lamproderma*, followed by *Didymium* (Fig. 1B). Since species of *Lamproderma* are mostly not amenable to cultivation, in contrast to *Didymium*^58^, it is likely that the abundance of the latter was overestimated by MPN methods. The wide occurrence of *Lamproderma* in soils is backed by previous molecular sampling results, as well as those of a fruiting bodies inventory conducted over 20 years^29^. The assemblage in our study was dominated by sequences related to the large *L. scintillans* clade (Fig. S2), a species complex of a world-wide occurrence (http://www.discoverlife.org/20/q, last accessed October 2014).

The assemblages of *Acanthamoeba* were dominated by genotypes T4 and T2 in every exploratory, totaling 85.4% of the OTUs. T4 has been reported to be the most abundant and widespread acanthamoebal genotype in all types of environments, with isolates from Asia, Europe and North America^43^ and it has functional significance as a potential human pathogen provoking eye infections (*Acanthamoeba* keratitis)^39^,^59^,^60^. It was the most common in grasslands soils in Italy, the Netherlands and Tibet^36^. A closer observation of the T4 sequences revealed a wide range of structures and lengths in the variable helices, suggesting that the current genotype concept in *Acanthamoeba*, based on sequences similarities in the conserved regions of the SSU rDNA gene is far too conservative. Accordingly, our phylogenetic tree shows that T4 is composed of several distinct genotypes, sometimes intermingled with others (Fig. S1); this being consistent with current phylogenies^39^,^42^,^60^. The taxonomic confusion (arbitrary species names) and the inconsistent and too broad criteria for genotyping (especially the polyphyletic T4) is currently the major hindrance to establishing a biogeography of the genus *Acanthamoeba*.

Our results follow the general concept of the rare biosphere: the diversity of protistan OTUs was composed of a majority of ‘rare’ taxa and few common ones^61^, although this pattern is less pronounced in the species-poor *Acanthamoeba* assemblage. This is not uncommon in assemblages of few species^62^. However, the marked differences in OTU composition between and within our three sampling regions suggest distinct geographical distributions, as it has already been hypothesized for protists^25^,^63^ (Table 3, Fig. 6). Moreover, the community composition patterns were not random. Comunities with few OTUs were subsets of large ones, as confirmed by nestedness analyses, both in species composition and in occupancy of sites (Table 4).

The distinct communities could possibly correlate with land use intensity or physico-chemical soil characteristics and plant, fungal and bacterial abundance and diversity - a collaborative task that we are currently undertaking in the framework of the Biodiversity Exploratories. As a corollary, it suggests that the global protistan biodiversity assessed with “universal primers” is consistently excluding ubiquitous, dominant, and functionally important taxa. To be comprehensive, environmental PCR should also include primers specifically targeting dark-spored Myxomycetes, *Acanthamoeba* and other amoebozoans. In addition, the amoebozoan reference database is in urgent need to be augmented with sequences from well-identified specimens, and *Acanthamoeba* is in need of a comprehensive systematic revision.

## Methods

### Study sites and soil sampling

The three German Biodiversity Exploratories are the Biosphere Reserve Schorfheide-Chorin (SEG) in the State of Brandenburg, the National Park Hainich and its surroundings in the State of Thuringia (HEG) and the Biosphere Reserve Schwäbische Alb (AEG) in the State of Baden-Württemberg^44^. Each exploratory comprises 50 grassland sites from extensive pastures to highly fertilized meadows. Each site contains a study plot of 20 × 20 m. From all study plots, 150 soil samples were collected in a coordinated joint sampling within 14 days in April 2011. From each plot 14 soil cores of 8.3 cm diameter were taken every 3 m along two transects of 20 m each, oriented North-South and East-West, employing a soil corer. The surface layer (0–10 cm) was collected, after removing plants, pebbles and conspicuous roots. Soil cores from each plot were sieved (2 mm mesh size), mixed, homogenised and immediately frozen for further analysis.

### DNA extraction, amplicon libraries preparation and pyrosequencing

Soil DNA was extracted using the Power Soil DNA isolation kit (Mo Bio, Carlsbad, CA) following the manufacturer’s protocol. Primers were designed to target *Acanthamoeba* and Myxomycetes (Table 5). We used a two-step approach to obtain amplicons, using 1 μl of soil DNA as a template for the first PCR and 1 μl of amplicon as a template for a following semi-nested PCR, using GreenTaq (Fermentas, Canada) (Table 5). To reduce artificial dominance of some PCR products, each sample was amplified twice and amplicons pooled together. Amplicons were cut from electrophoresis gels and purified using the GelElute Extraction Kit (5Prime, Hamburg, DE), their concentration measured with a spectrophotometer. In total, we obtained 146 final amplicons that were divided into 4 libraries, of 37 (2x) and 36 samples (2x), using only 37 different sample-specific barcodes (attached to the primers used for the second PCR) (Table S4). Adaptators ligation and sequencing were performed on a Roche GS FLX+ System (GS XLR 70 sequencing-kit) (GATC Biotech AG, Konstanz, DE).

### Sequences processing and quality filtering

In total, 924,102 reads were obtained and separated into two sets, *Acanthamoeba* and Myxomycetes, according to the primers sequences. All reads with an average quality score of less than 25, containing any ambiguities (either in the barcode, the primer or the sequence) and shorter than 200 nucleotides were removed using Mothur v.1.29.2^64^, which was also used in the following steps. We removed reads containing homopolymers larger than 8 and 13 nucleotides in Myxomycetes and *Acanthamoeba* dataset, respectively; the latter because at least two taxa, *Acanthamoeba tubiashi* AF019065 and *A. astronyxis* AF01906412 possess a 12-T polymer in the amplified fragment. Reads were trimmed to 530 nucleotides. Reads generated by the reverse primers were excluded because they encompassed mainly a conserved region. Artificial duplicates (reads that begin at the same position but may vary in length or bear mismatches) are known to be generated by 454 sequencing^65^. We clustered them using cd-hit v.4.6.1^65^, with the following settings: clustering into the most similar cluster that met the threshold of 97%, with the longest sequence representing the cluster (accurate but slow mode) and a word length of 10. Each set was then separated, by the mean of the barcodes, into the three exploratories (AEG, HEG and SEG), resulting in six sets (three for *Acanthamoeba* and three for Myxomycetes) (Table 1). A set or the three exploratories together was also analysed in parallel, one for each taxon, to allow for inter-site comparison.

### OTUs identification by BLAST

We first compared our filtered reads with the Silva non-redundant SSU rDNA reference dataset (downloaded from http://www.arb-silva.de/download/arb-files/, last accessed 9.4.2013) using BLAST (Table 1). The full Silva SSU rDNA Ref 111 includes sequences with an alignment identity over 70%. Therefore, divergent protistan sequences are excluded, and indeed only 59 myxomycete sequences were present. We used the Silva database to identify *Acanthamoeba* sequences and to exclude contaminants from the myxomycete dataset. To precisely identify myxomycete sequences, we made a reference dataset from GenBank comprising 133 sequences of Stemonitida, Physarida and Echinosteliida (=dark spore Myxomycetes) (Supplementary information 5). Search of the best hit was conducted with BLAST^66^ implemented in BioEdit v. 7.1.11^67^ with an expectation value of 1.0e-05.

### Template alignments

Direct alignment of these highly variable sequences using MAFFT v.7^68^ produced overlong alignments, up to 10,000 positions. Since misaligned sequences can be an acute source of diversity overestimation^69^, we created template alignments by selecting representative environmental sequences, using MAFFT with the FFT-NS-i (Slow; iterative refinement method), a gap opening penalty of 3.0 and an offset value of 0.1, and then refined by hand (Supplementary information 6 and 7). Environmental sequences were aligned to the template using Mothur (gap-opening penalty = 5), then refined by hand using BioEdit. Sequences that were too short (starting after five positions and ending before 90% of the sequences do) were removed (Table 1).

### Chimera detection

Chimeras are artefactual sequences composed of two distinct amplicons and can represent 15 to 60% of the reads, increasing with species diversity^70^. For better detection, we used two complementary approaches, first using UCHIME^71^ implemented in Mothur, using the aligned sequences as their own references, a minimum score of 0.5 and a minimum divergence ratio of 2.5; and a further manual check. For Myxomycetes (where the species diversity was higher) an additional step was taken, using the BLAST “identity”, i.e. the number of residues at the same positions in our sequences and their best hit: if the length of the fragment of our sequences matching a reference was <50% of its own total length, the sequence was removed.

### Operational taxonomic units (OTUs)

In order to avoid overestimation of the OTU richness due to remaining sequencing errors, we applied a low diversity threshold of 96% in clustering sequences into OTUs, using Mothur with the default settings (average neighbour algorithm), which has been shown to provide more reliable results in the presence of noise^72^,^73^ (Table 1). This threshold is even lower than the one that has been shown to faithfully reproduce a single bacterial genome (97%)^74^. Before clustering, we applied the preclustering method recommended by Huse *et al.* (2010), that takes into account read abundances to reduce the numbers of spurious OTUs (Table 1).

### Reference alignments and phylogenetic analyses

Two reference alignments were created for phylogenetic purposes. For *Acanthamoeba*, all Centramoebida sequences from the SSU rDNA Ref 111 database were aligned using MAFFT with the FFT-NS-i, a gap opening penalty of 3.0 and an offset value of 0.1. For dark spore Myxomycetes, we used the alignment described above (Supplementary information 5), without the Echinosteliida. The alignments were truncated to match our reads; void sequences, non-unique sequences and sequences with insufficient overlap were removed. Remaining sequences were clustered with a threshold of 96% similarity, resulting in 48 *Acanthamoeba* sequences, representing the 17 genotypes described until April 2013 and in 104 dark-spore myxomycete sequences. The alignments were checked and refined by hand, taking into account the positions of the variable helices 9, 10 and 10e1, following the secondary structure^32^,^40^,^75^; when possible, a tentative secondary structure was inferred for the acanthamoebal variable helices using the RNAalifold Web server (http://rna.tbi.univie.ac.at/cgi-bin/RNAalifold.cgi, last accessed August 2014)^76^. Environmental OTUs were aligned to the reference alignments using MAFFT as described above and refined by hand. The *Acanthamoeba* alignment totalled 321 sequences (48 reference sequences and 273 environmental OTUs) and 238 positions, of which 12% without polymorphism; it was characterized by very high relative T content (32%) and C<->T relative rate of substitution (11.4, according to the model below) (Supplementary information 8). The Myxomycete alignment totalized 442 sequences (104 reference sequences and 338 environmental OTUs) and 355 positions of which 18% without polymorphism (Supplementary information 9). Phylogenetic analyses were run using PhyML v. 3.0^77^ with the GTR + γ (4 categories) model of nucleotide substitution and the default parameters, including an approximate likelihood branch support estimation. (Figs S1 and S2).

### Statistical analyses

Statistical analyses were conducted on community matrices of OTUs (Table S4). Individual-based rarefaction curves were constructed to evaluate the sequencing depth using Mothur with a re-sampling of 1,000 randomizations without replacement. Beta-diversity based analyses were conducted on matrices of relative abundance, comparing the total distribution of the OTUs with their distribution in each region. To estimate infra-site similarities we used the multiple-site Simpson-based Index, with the R script provided^78^, discarding the samples with lesser sequences (<quartile) that is the first 11 samples having respectively less than 35 (AEG), 75 (HEG) and 129 (SEG) myxomycete sequences, and the first 10 (AEG) and 12 (HEG and SEG) samples having less than four, 550 and 230 acanthamoebal sequences, respectively. Inter-exploratories diversity was estimated using Mothur (Venn diagrams). Differences in community composition between regions were analyzed by a principal coordinates analysis (PCoA) using the R vegan package^79^ with Hellinger-transformed OTUs abundances of the three exploratories, with only OTUs > 11 sequences, resulting in 208 OTUs (124 *Acanthamoeba* and 84 Myxomycetes) (Table S4). Significance was tested using the envfit function of the R vegan package^79^ with 999 permutations. Permutational multivariate analyses of variance (PerMANOVA) and analyses of similarities (ANOSIM) were performed using adonis and anosim R scripts (vegan package)^79^, based on the Bray-Curtis community dissimilarity index with 10,000 permutations and the three exploratories as factors. To investigate the metacommunity stucture and identify non-random patterns of species compositions, we calculated the community nestedness using binary matrices, for the three exploratories together and for each of the three exploratories. The NeD software^80^ was run to calculate two different metrics, NODF and BR, with the following parameters: reliability assessed by Z values computed using 100 generated null matrices with proportional row and column totals.

## Additional Information

**How to cite this article**: Fiore-Donno, A. M. *et al.* Metacommunity analysis of amoeboid protists in grassland soils. *Sci. Rep.* **6**, 19068; doi: 10.1038/srep19068 (2016).

## Supplementary Material

Supplementary Information

Supplementary Table S4

Supplementary Information 5

Supplementary Information 6

Supplementary Information 7

Supplementary Information 8

Supplementary Information 9

## Figures and Tables

**Table 1:** Reduction and clustering of reads during the quality filtering. Initial number of reads = 924,102, of which 81% were further excluded. The unique BLAST hits give a rough estimation of the diversity.

	Trimmed	De-replication (cd-hit)	Validated by BLAST (Silva)	Second BLAST (our database)	Unique BLAST hits	Aligned, short sequences removed	Chimera removal (uchime) & by eye	Pre-cluster (mothur)	OTUs 96% sim.	Unique BLAST hits	# of sequences
*A Acanthamoeba*											
AEG	29,144	619	555		50	396	377	309	87	28	28,224
HEG	59,094	847	778		52	535	496	398	102	26	56,176
SEG	46,318	855	732		54	239	216	191	84	27	40,916
TOTAL	134,556	2,321	2,065		55	1,170	1,089	898	273	36	125,316
Clustered or removed		98%	11%			43%	7%	18%	70%		
B Myxomycetes											
AEG	7,392	1,074	971	899	46	892	500	349	100	24	4,763
HEG	13,460	1,221	1,146	1,095	51	1,039	587	387	111	31	9,606
SEG	21,874	1,106	989	897	51	795	353	290	127	31	14,960
TOTAL	42,726	3,401	3,106	2,891	60	2,726	1,440	1,026	338	35	29,329
Clustered or removed		92%	9%	7%		6%	47%	29%	67%		

**Table 2:** Taxonomic assignment of the contaminant reads.

	Clustered reads	*Contaminants*					Undetermined reads	Genuine reads
		Fungi	Alveolata	Other Amoebozoa	Other eukaryotes	Total		
*Acanthamoeba*	2,321	44	27	117	27	215 (9%)	41 (2%)	2,065 (89%)
Myxomycetes	3,401	82	186	15	12	295 (9%)	215 (6%)	2,891 (85%)
Total	5,722	126	213	132	39	510 (9%)	256 (4%)	4,956 (87%)

**Table 3:** Multiple-site Simpson-based similarity indices calculated for each exploratory.

	AEG	HEG	SEG
***Acanthamoeba***	0.3677	0.298	0.21
**Myxomycetes**	0.1151	0.1249	0.1426

**Table 4:** Nestedness of *Acanthamoeba* and Myxomycetes OTUs per region and per site. The sites are in rows and the OTUs in columns. Nestedness (p-value < 0.05) is indicated by numbers in bold.

	Nestedness								Matrix characteristics			
	NODF	Z-score	NODF/row	Z-score	NODF/col	Z-score	BR	Z-score	OTUs (col)	Plots (rows)	Occurrences	% of presence
A *Acanthamoeba*												
All	**46**	48	**61**	63	**38**	34	**1,710**	−29	184	136	4,599	18
AEG	**76**	16	**80**	15	**76**	16	**171**	−22	85	29	1,097	45
HEG	**73**	14	**82**	18	**72**	13	**229**	−22	95	36	1,718	50
SEG	**66**	15	**74**	18	**65**	13	**276**	−15	80	37	1,211	41
B Myxomycetes												
All	**26**	37	**43**	55	**19**	22	**1,101**	−15	203	130	2,225	8
AEG	**34**	8	**49**	14	**33**	6	**230**	−9	91	33	580	19
HEG	**33**	9	**50**	16	**32**	7	**257**	−10	105	35	668	18
SEG	**42**	13	**57**	22	**40**	12	**293**	−13	117	33	818	21

NODF values are comprized between 0 (no nestedness) and 100 (maximum nestedness), Z values > 1.64 indicate significance at p = 0.05.

BR values increase with nestedness, Z values < 1.64 indicate significance at p = 0.05.

Nestedness among rows = species composition.

Nestedness among columns = species occupancy of sites.

**Table 5:** Primers and PCR settings used in this study.

Name	sequence 5′-3′	specificity	reference
*First PCR, forward*^1^			
S1	AACCTGGTTGATCCTGCC	non-specific	Fiore-Donno *et al.* (2008)
*First PCR, reverse*			
SRAca28	CCAATTACAAGACTCTTRTCGAG	*Acanthamoeba*	this work
SR19Dark	GTCCTCTAATTGTTACTCGAD	dark-spored Myxomycetes	this work
*Second, semi-nested PCR, forward (reverse primers as before)*^2^			
SFAca22	CGGYGAGACTGCGGATGG	*Acanthamoeba*	this work
SF2Dark	GTTGATCCTGCCAGTAGTGT	dark-spored Myxomycetes	this work

^1^40 cycles, 30 s at 95 °C, 60 s at 54.8 °C, 120 s at 72 °C, and a final elongation step of 5 min at 72 °C.

^2^35 cycles, 30 s at 95 °C, 60 s at 54 °C, 120 s at 72 °C, and a final elongation step of 5 min at 72 °C.
