# Supplementary material for: Metacommunity analysis of amoeboid protists in grassland soils
Source: Sci Rep. 2016 Jan 11;6:19068. doi: 10.1038/srep19068 (PMC4707496; doi:10.1038/srep19068)
Supplement: Supplementary Information [file srep19068-s1.pdf]

Metacommunity analysis of amoeboid protists in grassland soils
Fiore-Donno AM, Weinert J, Wubet T & Bonkowski M.

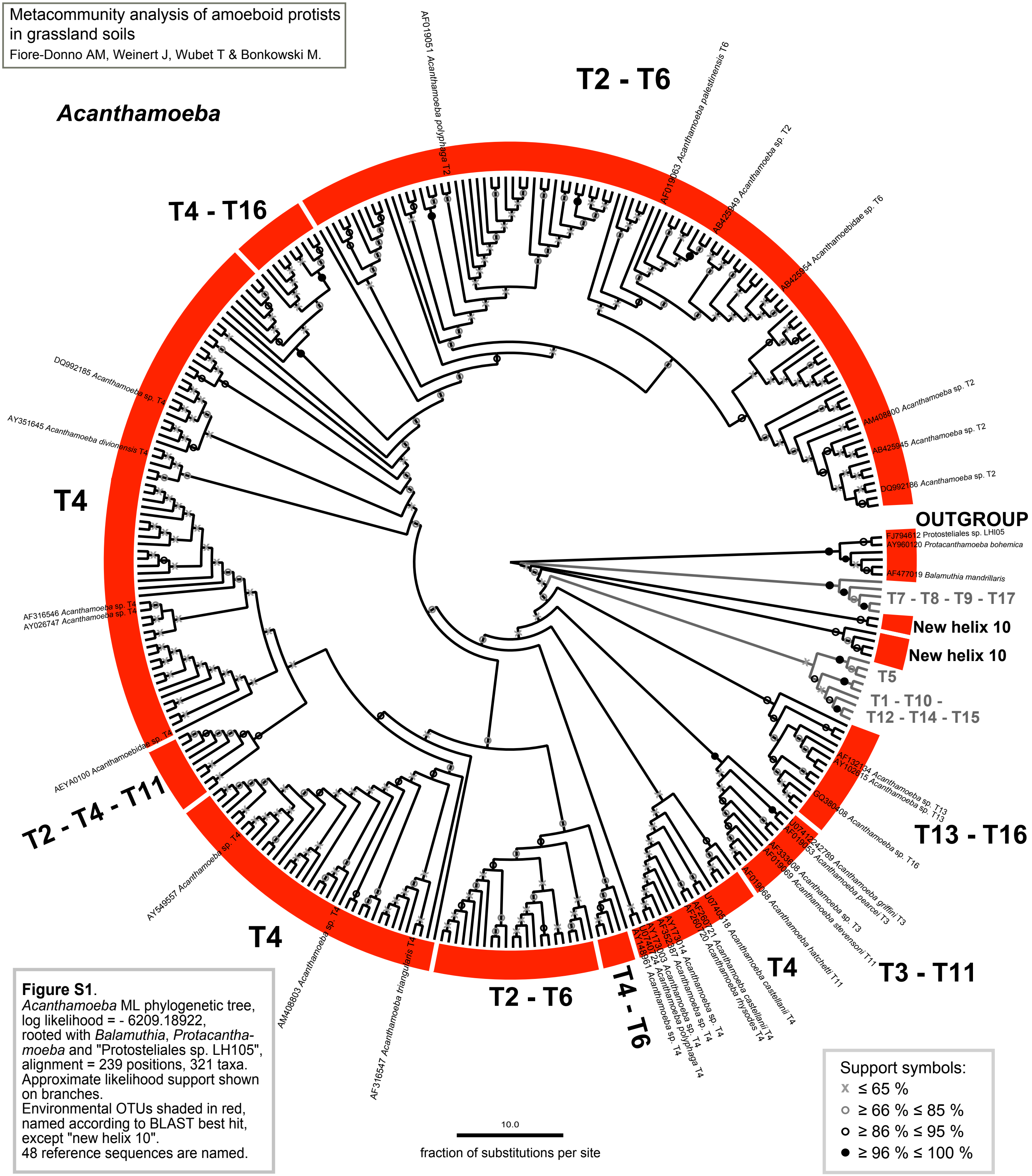

Figure S1.
Acanthamoeba ML phylogenetic tree, log likelihood = - 6209.18922, rooted with *Balamuthia*, *Protacanthamoeba* and "Protosteliales sp. LH105", alignment = 239 positions, 321 taxa. Approximate likelihood support shown on branches.
Environmental OTUs shaded in red, named according to BLAST best hit, except "new helix 10".
48 reference sequences are named.

Metacommunity analysis of amoeboid protists in grassland soils

Fiore-Donno AM, Weinert J, Wubet T & Bonkowski M.

MYXOMYCETES

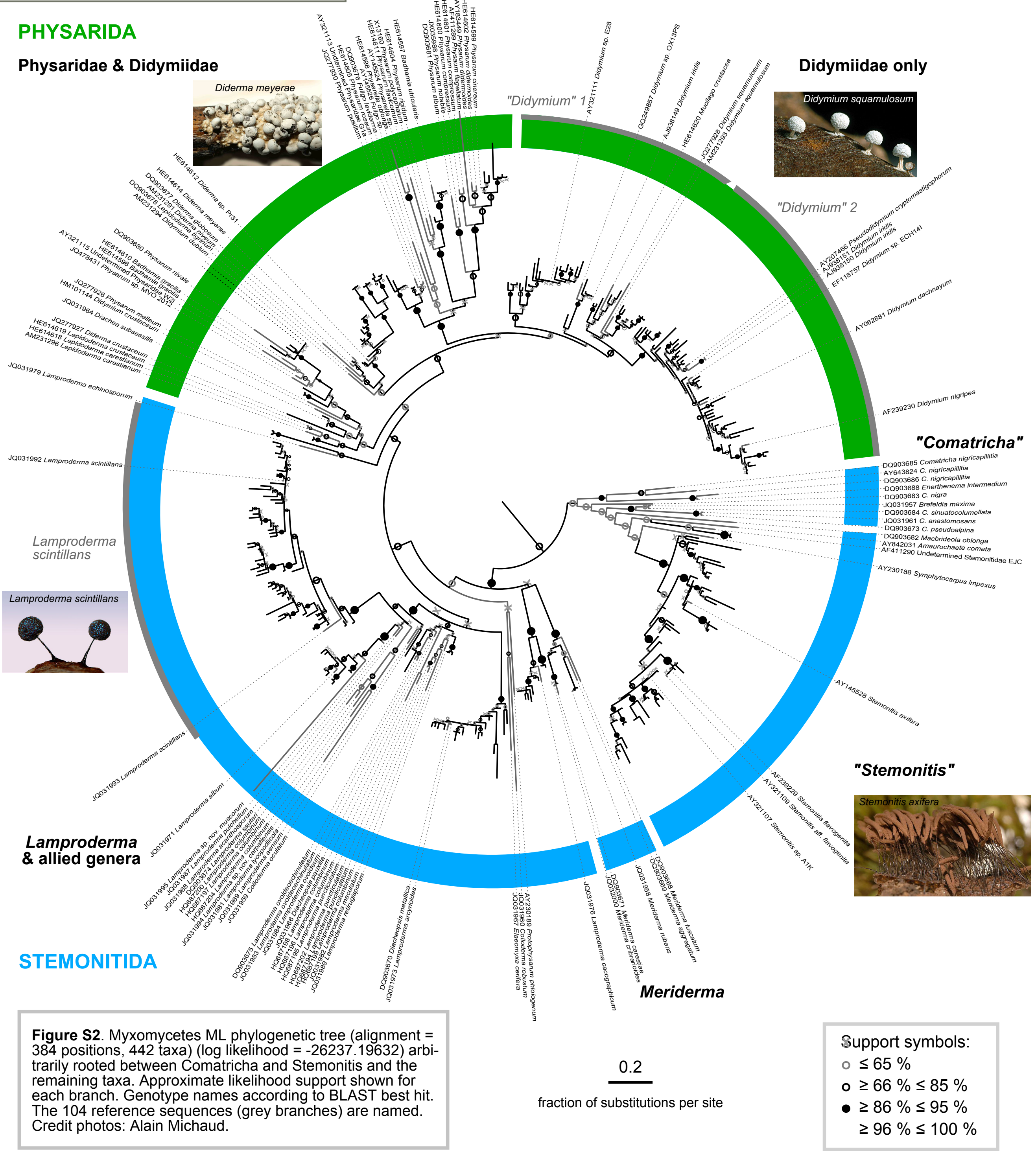

Figure S2. Myxomycetes ML phylogenetic tree (alignment = 384 positions, 442 taxa) (log likelihood = -26237.19632) arbitrarily rooted between Comatricha and Stemonitis and the remaining taxa. Approximate likelihood support shown for each branch. Genotype names according to BLAST best hit. The 104 reference sequences (grey branches) are named. Credit photos: Alain Michaud.

Metacommunity analysis of amoeboid protists in grassland soils

Fiore-Donno AM, Weinert J, Wubet T & Bonkowski M.

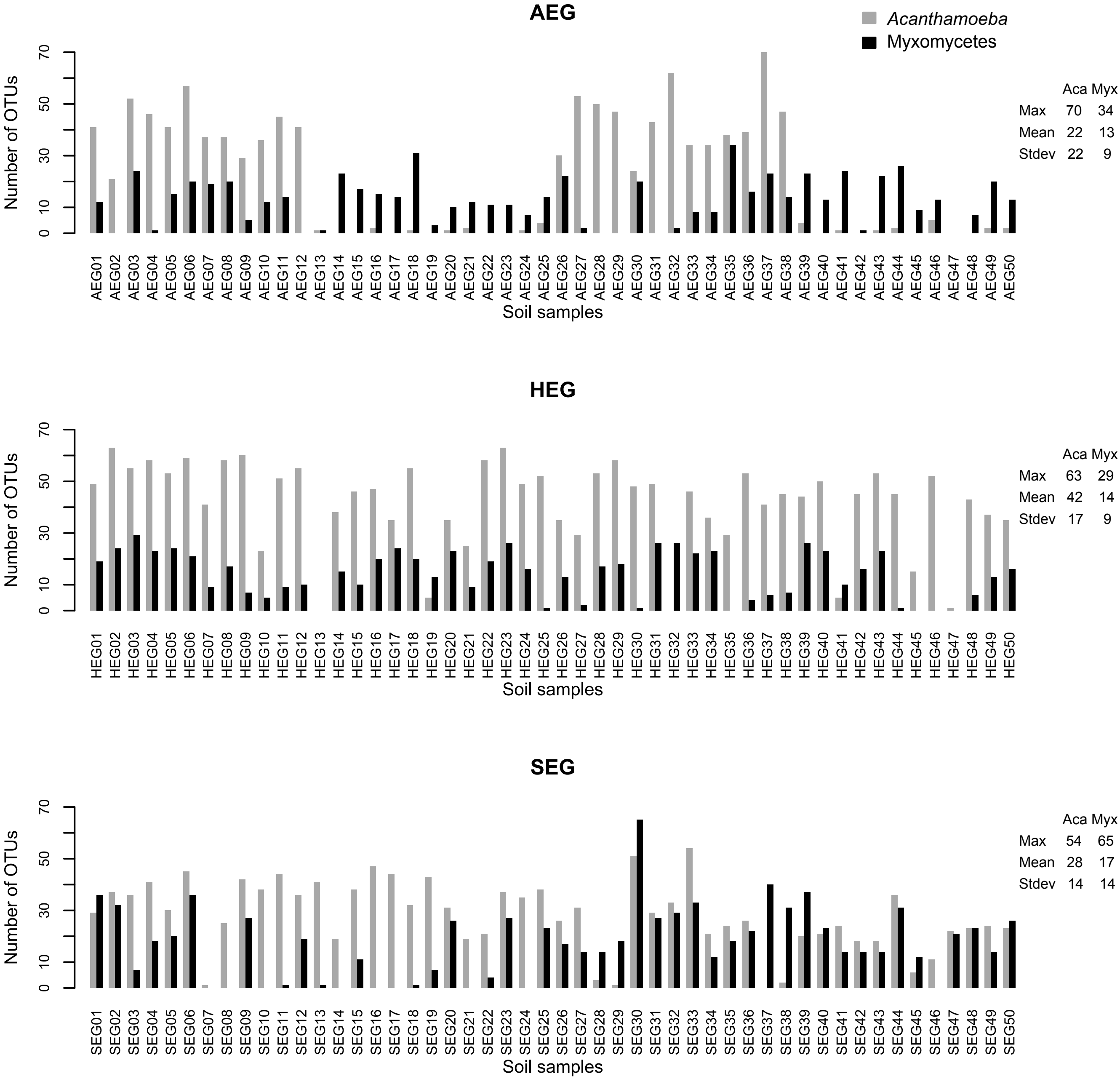

Figure S3. Number of OTUs per soil samples in each exploratory. The two taxa are shown side by side.
No clear pattern of co-occurrence between the two taxa can be seen. The higher number of acanthamoebal OTUs per site is explained by the higher number of shared OTUs between sites, when compared to the more diverse Myxomycetes.
